# Fluid challenge in critically ill patients receiving haemodynamic monitoring: a systematic review and comparison of two decades

**DOI:** 10.1186/s13054-022-04056-3

**Published:** 2022-06-21

**Authors:** Antonio Messina, Lorenzo Calabrò, Luca Pugliese, Aulona Lulja, Alexandra Sopuch, Daniela Rosalba, Emanuela Morenghi, Glenn Hernandez, Xavier Monnet, Maurizio Cecconi

**Affiliations:** 1 Department of Anesthesia and Intensive Care Medicine, Humanitas Clinical and Research Center—IRCCS, Via Alessandro Manzoni, 56, 20089 Rozzano, Milano Italy; 2 Department of Biomedical Sciences, Humanitas University, Pieve Emanuele, Milano Italy; 3 Università del Piemonte Orientale, Vercelli, Italy; 4 Departamento de Medicina Intensiva, Facultad de Medicina, Pontificia Universidad Católica de Chile, Diagonal Paraguay 362, Santiago, Chile; 5 Hôpitaux Universitaires Paris-Sud, Hôpital de Bicêtre, Medical Intensive Care Unit, Le Kremlin-Bicêtre, F-94270 Paris, France; 6 Université Paris-Saclay, AP-HP, Service de médecine intensive-réanimation, Hôpital de Bicêtre, Paris, France; 7 DMU CORREVE, Inserm UMR S_999, FHU SEPSIS, Groupe de Recherche Clinique CARMAS, Le Kremlin-Bicêtre, Paris, France

**Keywords:** Fluids, Fluid challenge, Fluid bolus, Fluid responsiveness, Critically ill patients

## Abstract

**Introduction:**

Fluid challenges are widely adopted in critically ill patients to reverse haemodynamic instability. We reviewed the literature to appraise fluid challenge characteristics in intensive care unit (ICU) patients receiving haemodynamic monitoring and considered two decades: 2000–2010 and 2011–2021.

**Methods:**

We assessed research studies and collected data regarding study setting, patient population, fluid challenge characteristics, and monitoring. MEDLINE, Embase, and Cochrane search engines were used. A fluid challenge was defined as an infusion of a definite quantity of fluid (expressed as a volume in mL or ml/kg) in a fixed time (expressed in minutes), whose outcome was defined as a change in predefined haemodynamic variables above a predetermined threshold.

**Results:**

We included 124 studies, 32 (25.8%) published in 2000–2010 and 92 (74.2%) in 2011–2021, overall enrolling 6,086 patients, who presented sepsis/septic shock in 50.6% of cases. The fluid challenge usually consisted of 500 mL (76.6%) of crystalloids (56.6%) infused with a rate of 25 mL/min. Fluid responsiveness was usually defined by a cardiac output/index (CO/CI) increase ≥ 15% (70.9%). The infusion time was quicker (15 min vs 30 min), and crystalloids were more frequent in the 2011–2021 compared to the 2000–2010 period.

**Conclusions:**

In the literature, fluid challenges are usually performed by infusing 500 mL of crystalloids bolus in less than 20 min. A positive fluid challenge response, reported in 52% of ICU patients, is generally defined by a CO/CI increase ≥ 15%. Compared to the 2000–2010 decade, in 2011–2021 the infusion time of the fluid challenge was shorter, and crystalloids were more frequently used.

**Supplementary Information:**

The online version contains supplementary material available at 10.1186/s13054-022-04056-3.

## Introduction

Fluid administration in the intensive care unit (ICU) is one of the most common and disputed interventions triggered at the bedside by several clinical variables [1, 2]﻿.

Fluid therapy aims to increase stroke volume (SV) and cardiac output (CO) to optimise systemic blood flow and tissue perfusion. As with any therapeutic intervention, the final clinical effect elicited may vary because of a complex interplay between the patient's intrinsic conditions and the therapy itself.

Fluid responsiveness can occur only if both ventricles work on the ascending, steep part of the Frank–Starling curve, i.e. in cases where CO is preload dependent [3, 4]. Preload dependency is assessed using a diagnostic test performed by infusing a fixed aliquot of fluid, the fluid challenge [5–7]. From a clinical perspective, this approach also allows titration of fluid administration (when the patient becomes no longer responsive to the fluid challenge) and reduces the risk of fluid overload, which worsens the outcome of ICU patients [8, 9].

Several variables defining the characteristics of the fluid challenge have been further investigated in studies adopting continuous haemodynamic monitoring, showing that the amount of fluids given, the rate of administration, and the threshold adopted to define fluid responsiveness impact the outcome of a fluid challenge [10–12]. Moreover, despite conflicting results on shock reversal efficacy between crystalloids and colloids, crystalloids are now recommended as the first-line fluid type in patients with septic shock, being inexpensive and widely available. Also, the administration of colloids compared to crystalloids has not demonstrated any clear benefit in the literature [13, 14].

However, neither the nature, mode of administration, and method to assess the effectiveness of the fluid challenge are standardised in current clinical practice, and the definition of fluid challenge responsiveness is also variable among studies [15–18].

Whether or not these findings have modified the modalities of fluid challenge and the definition of fluid responsiveness in published studies is uncertain. To address this issue, we systematically reviewed existing literature from the year 2000. We appraised the characteristics of fluid challenges in critically ill patients (i.e., amount and kind of fluid administration, time of infusion, hemodynamic variables, and thresholds for fluid responsiveness) enrolled in research studies receiving continuous haemodynamic monitoring and assessed the relationship between the reported fluid responsiveness and predefined independent variables. Secondarily, we compared data from studies published in 2011–2021 versus those published in 2000–2010.

## Material and methods

We adhered to the *Preferred Reporting Items for Systematic Reviews and Meta-Analysis-Protocols* (PRISMA-P) guidelines (Additional file 1: Table S1). The study protocol was registered with the *International Prospective Register of Systematic Reviews* (PROSPERO) in November 2021 (CRD42021284761).

### Search strategy

A systematic literature search was performed, including the following databases: PUBMED®, EMBASE®, and the Cochrane Controlled Clinical trials register. The following keywords and their related MeSh terms were used: “fluid challenge”, “fluid responsiveness”, “stroke volume variation”, “pulse pressure variation”, “dynamic indices OR indexes”, “passive leg raising”, OR “passive leg raising test”, “functional haemodynamic test OR tests”. Included papers were also examined to identify other studies of interest missed during the primary search.

### Study selection and inclusion criteria

Articles enrolling at least 20 adult critically ill patients, written in English and published from 1st January 2000 to 31st December 2021 in indexed scientific journals, were considered. Editorials, commentaries, letters to the editor, opinion articles, reviews, and meeting abstracts were excluded. Studies enrolling paediatric or obstetric populations were excluded. References of selected papers, review articles, commentaries, and editorials on this topic were also reviewed to identify other studies of interest missed during the primary search. When multiple publications of the same research group/centre described potentially overlapping cohorts, the most recent publications were selected.

A fluid challenge was defined as an infusion of a definite quantity of fluid (expressed as a volume in mL or ml/kg) in a fixed time (expressed in minutes), whose outcome was defined as a change in one of the following haemodynamic variables above a predetermined threshold: CO, cardiac index (CI), SV, SV index (SVI), or surrogate of SV, i.e., velocity–time integral (VTI) in the left ventricular outflow tract and aortic blood flow (ABF), as assessed by transthoracic, transoesophageal echocardiography or oesophageal Doppler. We included studies adopting both a specific (i.e., Ringer lactate, saline, etc.) and a broad definition (i.e., crystalloids, colloids, etc.) of the fluid used for the fluid challenge. Studies adopting changes in systemic arterial pressure to define fluid responsiveness were excluded. Finally, we considered the predefined clinical reasons and triggers to start fluid challenge infusion.

### Data extraction

Three couples of examiners independently evaluated titles and abstracts. The articles were then subdivided into three subgroups: “included” and “excluded” (if the two examiners agreed with the selection) or “uncertain” (in case of disagreement). In the case of “uncertain” classification, discrepancies were resolved by further examination performed by one of the three expert authors (A.M., X.M., or M.C.). We used a standardised electronic spreadsheet (Microsoft Excel, V 14.4.1; Microsoft, Redmond, WA) to extract data from all included studies, recording: the study setting (type of study, geographical area and time, where and when the study was carried out, and sample size), patient characteristics (gender, age, reason for admission, underlying diseases, ICU scores of severity, mode of ventilation, and inotropic/vasopressor support), criteria for haemodynamic instability, fluid challenge characteristics, pre- and post-fluid challenge haemodynamic variables. When necessary, the corresponding authors of the included studies were contacted to obtain missing data related to trial demographics, methods, and outcomes (Additional file 1: Table S2).

### Statistical analysis

Statistical analysis was conducted on the summary statistics described in the selected articles (e.g., means, medians, proportions) and, therefore, the statistical unit of observation for all the selected variables was the single study and not the patient. Due to the discrepancy between the overall patients enrolled in the trials over the two considered decaders, the comparisons were not weighted for study size.

Fluid challenge was the exposure variable, and clinical and haemodynamic characteristics were considered outcome variables. Descriptive statistics of individual studies used different statistical indicators for central tendency and variability, such as means and standard deviations (i.e., age, tidal volume, fluid responders, severity scores), whereas absolute and relative frequencies were adopted for qualitative variables. To show one indicator for the quantitative variables, we collected means with standard deviations (SD) or medians and inter-quartile ranges (IQR).

Student's t test or Mann–Whitney test in case of parametric or nonparametric distributions, respectively, were used to assess the difference in mean values between responders and non-responders.

Statistical analyses were conducted using GraphPad PRISM® 8 (GraphPad Software Inc., San Diego, CA, USA) and STATA®15 (StataCorp, College Station, TX, USA). For all comparisons, we considered *p* values < 0.05 significant.

## Results

**Fig. 1:**
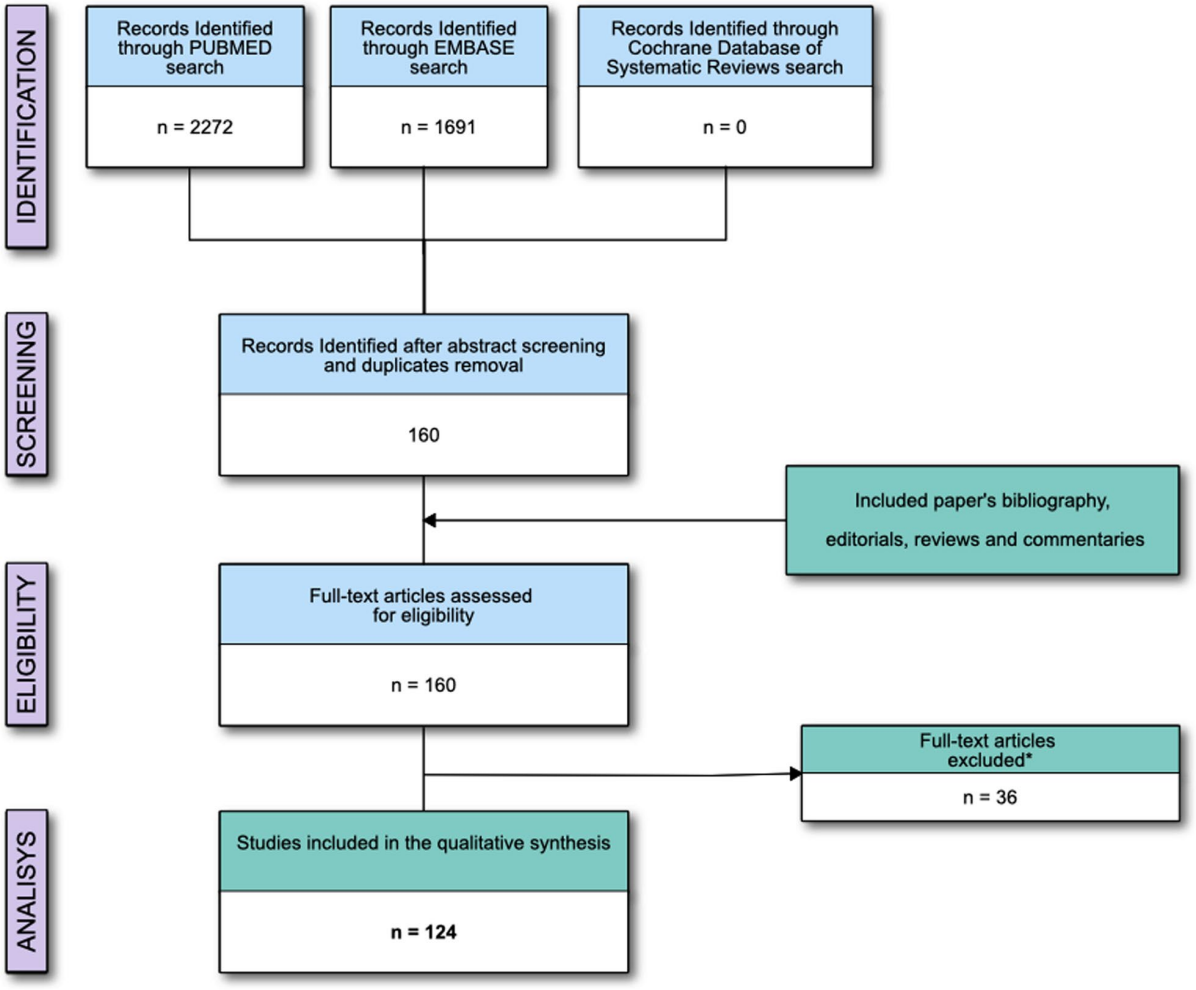
Flow of the studies; *Reasons for studies' exclusion are reported in the Supplementary materials

The electronic search identified 3,963 potentially relevant studies. Figure 1 and Additional file 1: Table S3 provide a detailed description of the selection process flow. After evaluating 160 full-text manuscripts, the inclusion criteria were met by 124 studies, 32 (25.8%) published in the period 2000–2010 and 92 (74.2%) in the period 2011–2021. Ten studies (8.1%) required revision by senior examiners because of disagreement regarding inclusion criteria between the initial examiners. We did not find any further relevant publications by reviewing the bibliography of the selected studies.

The general characteristics of the patients are reported in Table 1. We included 6,086 patients, with a median (IQR) of 38 (30–59) patients enrolled in each study. Six studies (4.8%) [20–25] were retrospective, while the others were prospective. The median (IQR) period of enrolment [reported in 66 (52.8%) studies] was 12 (6–18) months. At baseline, 2,985 (49.0%) patients received norepinephrine, 179 (2.9%) dopamine, 416 (6.8%) dobutamine, and 177 (2.8%) epinephrine.

**Table 1:** Patient characteristics at enrolment

General characteristics	Overall (n = 6086)	% DR	2011–2021 (n = 1243)	2000–2010 (n = 4843)	*p* value**
Age (year)	63 (59–65)	(95.9)	63 (59–65)	63 (58–67)	0.52
Male (n, %)	3552 (58.3)	(87.1)	2837 (58.6)	715 (57.5)	0.52
SAPS II (points)	53 (45–59)	(39.5)	55 (45–60)	53 (40–57)	0.34
SOFA (points)	10 (7–11)	(22.5)	10 (7–11)	11 (9–14)	0.10
APACHE (points)	20 (19–26)	(22.5)	24 (19–27)	19 (12–21)	0.12
**Data regarding respiratory support**		(70.9)			
*Totally controlled ventilatory support (n; %)*	*2658 (56.6)*		*1955 (56.2)*	*703 (57.9)*	0.29
PCV (n; %)	150 (3.2)		128 (3.6)	22 (1.8)	
VCV (n; %)	2428 (51.7)		1747 (50.2)	681 (56.1)	
APRV (n; %)	80 (1.7)		80 (2.3)	0 (0–0)	
*Partially controlled ventilatory support (n; %)*	*1503 (32.0)*		*1161 (33.4)*	*342 (28.1)*	0.05
ACV (n; %)	1256 (26.7)		990 (28.4)	266 (21.9)	
PSV (n; %)	247 (5.3)		171 (4.9)	76 (6.2)	
*Spontaneously breathing (n; %)*	*530 (10.1)*		*362 (10.4)*	*168 (13.8)*	0.001
V_T_ (mL/Kg ideal body weight)	7 (7–8)	(70.9)	7 (6–8)	8 (7–9)	0.03
Type of patients*		(91.9)			
Sepsis/septic shock (%)	3546 (50.6)		2823 (50.5)	723 (51.2)	0.63
Haemorrhagic/hypovolemic shock (%)	344 (4.9)		252 (13.9)	92 (6.5)	< 0.0001
Trauma (%)	120 (1.7)		101 (1.8)	19 (1.3)	0.25
ARDS/pneumonia (%)	1741 (24.8)		1428 (25.5)	313 (22.2)	0.009
Postoperative optimization (%)	1005 (14.3)		780 (13.9)	225 (15.9)	0.06
Cardiogenic (%)	193 (2.7)		160 (2.8)	33 (2.3)	0.31
Other (%)	53 (0.7)		47 (2.8)	6 (0.5)	0.12

Data presented as median (25th–75th IQR), as appropriate; % DR, percentage of studies reporting the data indicated, SAPS, simplified acute physiology score; SOFA, sequential organ failure assessment; APACHE, Acute Physiologic Assessment and Chronic Health Evaluation; PCV, pressure-controlled ventilation; VCV, volume-controlled ventilation; APRV, Airway pressure release ventilation; ACV, assisted-controlled ventilation; PSV, pressure support ventilation; ARDS, acute respiratory distress syndrome; V_T_, tidal volume. *The overall number of patients stratified by typology is more significant as compared to the overall number of patients included in the studies because of partial overlapping classification (i.e., sepsis/septic shock and ARDS/pneumonia); **p value refers to the comparison between 2000–2010 versus 2011–2021 subgroups

The reliability of a functional haemodynamic test in predicting fluid responsiveness was assessed in 46 (37.1%) studies. Comparing the two considered decades, no difference was found in the rate of FC administration [17 min (17–33) vs. 33 min (17–50); *p* = 0.39), in the percentage of fluid responders [52% (43–67) vs. 53% (45–60); *p* = 0.91], in the percentage of studies adopting crystalloids over colloids [63.6% vs. 67.9%; *p* = 1.00), or in the threshold of increase in CO or surrogates adopted to define fluid responsiveness (10% over 15%) [18.2% vs. 24.1%; *p* = 1.00).

Forty-four studies (35.4%) investigated the reliability of a dynamic index in predicting fluid responsiveness. Comparing the two considered decades, no differences were found in the rate of FC administration [17 min (17–25) vs. 29 min (13–33) *p* = 0.42), or in the rate of fluid responders [53% (41–62) vs. 50% (44–56) *p* = 0.81), or in the threshold of increase in CO or surrogates adopted to define fluid responsiveness (10% over 15%) (78.5% vs. 66.67 *p* = 0.42), as compared to studies in the decade 2000–2010. On the contrary, in the decade 2010-2021 we adopted more frequently crystalloids (21.4% vs. 60.0% *p* = 0.024).

### Fluid challenge characteristics and haemodynamic monitoring

Overall, the included studies infused 6,333 fluid challenges. The median (IQR) proportion of fluid responders was 52 (44–62)% (Table 2).

**Table 2:** Fluid challenge characteristics and haemodynamic monitoring in the included studies

References	Year	Vol (ml)	Vol (ml/kg)	Time (min)	Rate (ml/min)	Responsiveness cut-off	Type of fluid	Monitoring device	% R
Mahjoub et al. [35]	2010	500	–	30	17	SV ≥ 15%	CRYS—Saline	CARDIOQ	76
Feissel et al. [37]	2004	500	–	20	25	CO ≥ 15%	COLL—HES 6%	ECO—TT/TE	41
Marik et al. [39]	2013	500	–	10	50	SVI ≥ 10%	CRYS—Saline	NICOM	53
Wyffels et al. [41]	2007	500	–	20	25	CI ≥ 15%	COLL—HES 6%	PAC	62
Jozwiak et al. [43]	2017	500	–	10	50	CI ≥ 15%	CRYS—Saline	CAL—PiCCO2	50
Monnet et al. [45]	2009	500	–	10	50	CI ≥ 15%	CRYS—Saline	CAL—PiCCO2	70
Monnet et al. [47]	2012	500	–	20	25	CI ≥ 15%	CRYS—Saline	CAL—PiCCO2	55
Vaquer et al. [20]	2020	500	–	30	17	SVI ≥ 15%	CRYS—Saline	CAL—PiCCO2	34
Chen et al. [49]	2021	500	–	40	13	CI ≥ 15%	COLL—HES 6%	CAL—PiCCO2	60
Abdullah et al. [49]	2021	500	–	10	50	SVI ≥ 15%	CRYS—Saline	UNCAL—FLOWTRAC/VIGILEO	46
Messina et al. [52]	2021	500	–	10	50	SVI ≥ 10%	CRYS—Ringer A/L	UNCAL—MOSTCARE	48
Taccheri et al. [54]	2021	500	–	10	50	CI ≥ 10%	CRYS—Saline	CAL—PiCCO2	50
Kaur et al. [56]	2021	500	–	20	25	CO ≥ 15%	CRYS—Ringer A/L	UNCAL—FLOWTRAC/VIGILEO	67
Biasucci et al. [58]	2019	500	–	30	17	CI ≥ 15%	COLL—HES 6%	PAC	60
Gavaud et al. [60]	2019	500	–	15	33	CO ≥ 10%	CRYS—Saline	ECO—TT/TE	90
Dépret et al. [62]	2019	500	–	10	50	CI ≥ 15%	CRYS—Saline	CAL—PiCCO2	50
Messina et al. [64]	2019	500	–	10	50	CI ≥ 15%	CRYS—Saline	UNCAL—MOSTCARE	66
Vistisen et al. [66]	2018	500	–	30	17	SV ≥ 10%	CRYS—Saline	NICOM	23
Xu et al. [68]	2017	500	–	15	33	CI ≥ 15%	COLL—Gelatine	PAC	45
Preau et al. [69]	2017	–	6	30	–	SVI ≥ 10%	COLL—Gelatine	CAL—PiCCO2	55
Machare-Delgado et al. [71]	2011	–	6	10	–	SVI ≥ 10%	CRYS—Saline	ECO—TT/TE	32
Monnet et al. [73]	2013	–	7	30	–	CI ≥ 15%	CRYS—Saline	CAL—PiCCO2	49
Monnet et al. [74]	2007	500	–	8	67	ABF ≥ 15%	CRYS—Saline	ECO—TT/TE	54
Ishihara et al. [76]	2013	250	–	20	13	CI ≥ 15%	COLL—Dextran 10%	CAL—PiCCO	54
Monge Garcia et al. [78]	2012	500	–	30	17	CO ≥ 15%	COLL—HES 6%	CARDIOQ	57
Luzi et al. [80]	2013	500	–	30	17	SV ≥ 15%	CRYS—Saline	ECO—TT/TE	50
Dong et al. [82]	2012	500	–	30	17	SVI ≥ 15%	COLL—HES 6%	CAL—PiCCO	69
Jabot et al. [84]	2008	–	20	10	–	CI ≥ 15%	CRYS—Saline	CAL—PiCCO	100
Préau et al. [86]	2010	500	–	30	17	SV ≥ 15%	COLL—HES 6%	ECO—TT/TE	41
Monnet et al. [88]	2006	500	–	10	50	ABF ≥ 15%	CRYS—Saline	ECO—TT/TE	52
Monnet et al. [47]	2012	500	–	20	25	CI ≥ 15%	CRYS—Saline	CAL—PiCCO	56
Monnet et al. [91]	2013	500	–	30	17	CI ≥ 15%	CRYS—Saline	CAL—PiCCO	43
Loupec et al. [93]	2011	500	–	10	50	CO ≥ 15%	COLL—HES 6%	ECO—TT/TE	53
Monnet et al. [95]	2012	–	8	30	–	CI ≥ 15%	CRYS—Saline	CAL—PiCCO	44
Huang et al. [97]	2008	–	7	40	–	CI ≥ 15%	COLL—HES 6%	CAL—PiCCO	46
Khwannimit et al. [98]	2012	500	–	30	17	SVI ≥ 15%	COLL—HES 6%	UNCAL—FLOWTRAC/VIGILEO	57
Fischer et al. [100]	2013	–	7	15	–	CI ≥ 15%	COLL—HES 6%	CAL—PiCCO	71
Kramer et al. [102]	2004	–	7	15	–	CO ≥ 15%	Blood	PAC	29
Yazigi et al. [104]	2012	–	10	20	–	SVI ≥ 15%	COLL—HES 6%	PAC	68
Wyler von Ballmoos et al. [106]	2010	200	7	10	20	SV ≥ 10%	COLL—HES 6%	PAC	28
Michard et al. [108]	2000	500	6	30	17	CI ≥ 15%	COLL—HES 6%	PAC	40
Lakhal et al. [110]	2011	500	–	30	17	CO ≥ 10%	COLL—Gelatine	PAC	40
Muller et al. [112]	2012	500	–	15	33	VTI ≥ 15%	COLL—HES 6%	ECO—TT/TE	50
Giraud et al. [114]	2011	500	–	10	50	CI ≥ 15%	CRYS—Saline	PAC	47
Suehiro et al. [116]	2012	500	–	30	17	CI ≥ 15%	CRYS—Ringer A/L	UNCAL—FLOWTRAC/VIGILEO	48
Perner et al. [117]	2006	–	4	30	–	CI ≥ 10%	COLL—Dextran 6%	CAL—PiCCO	47
Smorenberg et al. [118]	2013	250	–	15	17	SVI ≥ 10%	COLL—Gelatine	PAC	44
Monnet et al. [119]	2012	–	10	30	–	CI ≥ 15%	CRYS—Saline	CAL—PiCCO	42
Yonis et al. [121]	2017	500	–	15	33	CI ≥ 10%	CRYS—Saline	CAL—PiCCO	33
Xiao-ting et al. [122]	2015	500	–	15	33	CI ≥ 10%	CRYS—Saline	CAL—PiCCO	70
Biais et al. [124]	2009	500	–	15	33	SV ≥ 15%	CRYS—Saline	UNCAL—FLOWTRAC/VIGILEO	67
Mallat et al. [126]	2015	500	–	15	33	CI ≥ 15%	COLL—HES 6%	CAL—PiCCO	45
Maizel et al. [127]	2007	500	–	15	33	CO ≥ 15%	CRYS—Saline	ECO—TT/TE	50
Lamia et al. [129]	2007	500	–	15	33	SV ≥ 15%	CRYS—Saline	ECO—TT/TE	59
Silva et al. [131]	2004	500	–	30	17	CI ≥ 10%	COLL—HES 6%	PAC	63
Cecconi et al. [133]	2012	250	–	5	50	SV ≥ 15%	COLL—HES 6% /Dextran 10%	CAL—LiDCO	39
Georges et al. [135]	2018	500	–	15	33	CO ≥ 15%	CRYS—Saline	ECO—TT/TE	56
Monnet et al. [137]	2013	500	–	30	17	CI ≥ 15%	CRYS—Saline	CAL—PiCCO	52
Monnet et al. [139]	2005	500	–	10	50	VTI ≥ 15%	CRYS—Saline	ECO—TT/TE	53
Biais et al. [141]	2012	500	–	15	33	SV ≥ 15%	CRYS—Saline	UNCAL—MOSTCARE	54
Lakhal et al. [115]	2013	500	–	30	17	CO ≥ 10%	COLL—Gelatine	PAC	37
Michard et al. [144]	2003	500	–	30	17	SVI ≥ 15%	COLL—HES 6%	CAL—PiCCO	49

References	Year	Vol (ml)	Vol (ml/kg)	Time (min)	Rate (ml/min)	Responsiveness cut-off	Type of fluid	Monitoring device	% R
Préau et al. [36]	2012	–	6	30	–	SV ≥ 15%	COLL—HES 6%	ECO—TT/TE	44
Caille et al. [38]	2008	500	–	15	33	CI ≥ 10%	COLL—HES 6%	ECO—TT/TE	43
Mahjoub et al. [40]	2012	500	–	20	25	SV ≥ 15%	CRYS—Saline	ECO—TT/TE	71
Wu et al. [42]	2014	500	–	15	33	CO ≥ 15%	CRYS—Saline	ECO—TT/TE	54
Fellahi et al. [44]	2012	–	7	15	–	CI ≥ 15%	COLL—HES 6%	CAL—PiCCO	84
Smorenberg et al. [46]	2017	500	–	30	17	CO ≥ 10%	COLL—HES 6%	CAL—LiDCO	62
Muller et al. [48]	2011	500	–	15	33	VTI ≥ 15%	COLL—HES 6%	ECO—TT/TE	54
Monnet et al. [28]	2011	500	–	20	25	CI ≥ 15%	CRYS—Saline	CAL—PiCCO	62
Monge Garcia et al. [50]	2008	500	–	30	17	SVI ≥ 15%	CRYS—Saline	UNCAL—FLOWTRAC/VIGILEO	37
Natalini et al. [51]	2006	500	–	30	17	CI ≥ 15%	CRYS—Saline	PAC	59
Mahjoub et al. [53]	2009	500	–	30	17	SV ≥ 15%	COLL—Gelatine	ECO—TT/TE	66
Fischer et al. [55]	2013	–	7	15	–	CI ≥ 15%	COLL—HES 6%	CAL—PiCCO	73
Vistisen et al. [57]	2009	500	–	90	6	CI ≥ 15%	COLL—HES 6%	PAC	74
Kupersztych-Hagege et al. [59]	2013	500	–	10	50	CI ≥ 15%	CRYS—Saline	CAL—PiCCO	40
Monge Garcìa et al. [61]	2009	500	–	30	17	SVI ≥ 15%	COLL—HES 6%	UNCAL—FLOWTRAC/VIGILEO	50
Lakhal et al. [63]	2012	500	–	30	17	CO ≥ 10%	COLL—Gelatine	PAC	39
Soubrier et al. [65]	2007	500	–	20	25	CI ≥ 15%	COLL—HES 6%	ECO—TT/TE	59
Fellahi et al. [67]	2012	500	–	15	33	CI ≥ 15%	COLL—HES 6%	CAL—PiCCO	56
Osman et al. [22]	2007	500	–	20	25	CI ≥ 15%	COLL—HES 6%	PAC	43
Lakhal et al. [70]	2010	500	–	30	17	CO ≥ 10%	COLL—Gelatine	CAL—PiCCO	42
De Oliveira-Costa et al. [72]	2012	1000	–	30	33	CI ≥ 15%	CRYS—Saline	PAC	46
Velissaris et al. [23]	2011	500	–	30	17	CI ≥ 10%	COLL	PAC	52
Monnet et al. [75]	2011	500	–	10	50	CI ≥ 10%	CRYS—Saline	CAL—PiCCO	84
Muller et al. [77]	2010	500	–	30	17	SVI ≥ 10%	COLL—HES 6%	CAL—PiCCO	72
Muller et al. [79]	2008	500	–	30	17	SVI ≥ 15%	COLL—HES 6%	CAL—PiCCO	51
Heenen et al. [81]	2006	500	–	30	17	CO ≥ 15%	COLL—HES 6%	PAC	43
De Backer et al. [83]	2005	500	–	30	17	CI ≥ 15%	COLL—HES 6%	PAC	55
Le Dorze et al. [85]	2018	250	–	5	50	SV ≥ 10%	CRYS—Saline	CARDIOQ	35
Wu et al. [87]	2018	250	–	15	17	SVI ≥ 15%	CRYS—Saline	ECO—TT/TE	45
Si et al. [89]	2018	250	–	30	8	SVI ≥ 15%	COLL—Albumine	CAL—PiCCO	63
Pouska et al. [90]	2018	–	5	20	–	SVI ≥ 15%	CRYS—Saline	CAL—PiCCO2	49
Xu et al. [92]	2017	250	–	10	25	SV ≥ 10%	CRYS—Saline	NICOM	41
Soussi et al. [94]	2017	500	–	15	33	CI ≥ 15%	CRYS—Ringer A/L	CAL—PiCCO2	31
Mallat et al. [96]	2016	500	–	15	33	CI ≥ 15%	CRYS—Saline	CAL—PiCCO	52
Aya et al. [32]	2016	250	–	5	50	CO ≥ 10%	CRYS—Ringer A/L	CAL—LiDCO	50
Hamimy et al. [99]	2016	500	–	15	33	SV ≥ 10%	CRYS—Saline	CARDIOQ	89
Liu et al. [101]	2016	500	–	20	25	CO ≥ 15%	CRYS—Saline	CAL—PiCCO	54
Guérin et al. [103]	2015	500	–	10	50	CI ≥ 15%	CRYS—Saline	CAL—PiCCO2	50
Airapetian et al. [105]	2015	500	–	15	33	CO ≥ 10%	CRYS—Saline	ECO—TT/TE	49
Messina et al. [107]	2015	500	–	10	50	CI ≥ 15%	CRYS—Saline	UNCAL—MOSTCARE	–
Cecconi et al. [109]	2015	250	–	8	33	CO ≥ 10%	COLL—Gelatine	CAL—PiCCO	50
Soliman et al. [111]	2015	500	–	10	50	CI ≥ 15%	COLL—HES 6%	ECO—TT/TE	56
Nunes et al. [113]	2014	500	–	30	17	CI ≥ 15%	CRYS—Ringer A/L	PAC	65
Lakhal et al. [115]	2013	500	–	30	17	CO ≥ 10%	COLL—Gelatine	PAC	37
Monge García et al. [21]	2015	500	–	30	17	CO ≥ 10%	0,COLL—Gelatine	CARDIOQ	67
Cecconi et al. [26]	2013	250	–	5	50	CO ≥ 10%	–	CAL—LiDCO	43
Hu et al. [24]	2013	300	–	20	15	CI ≥ 10%	COLL—HES 6%	CAL—PiCCO	52
Schnell et al. [120]	2013	500	–	23	22	ABF ≥ 10%	CRYS—Saline	ECO—TT/TE	49
Pranskunas et al. [27]	2013	500	–	30	17	SV ≥ 10%	–	CAL—VIGILANCE	68
Elsayed et al. [123]	2021	–	4	15	–	CO ≥ 15%	CRYS—Ringer A/L	ECO—TT/TE	35
Bataille et al. [125]	2021	500	–	15	33	SV ≥ 15%	CRYS—Ringer A/L	ECO—TT/TE	50
De Santis et al. [25]	2021	500	–	30	17	CI ≥ 10%	–	CAL—PiCCO	58
Kumar et al. [128]	2021	–	10	30	–	CI ≥ 10%	CRYS—Saline	UNCAL—FLOWTRAC/VIGILEO	64
Braun et al. [130]	2020	500	–	15	33	SV ≥ 15%	CRYS—Ringer A/L	CAL—PiCCO	43
Huette et al. [132]	2020	500	–	10	50	SV ≥ 15%	CRYS—Ringer A/L	ECO—TT/TE	77
Abdelfattah et al. [134]	2020	500	–	15	33	CO ≥ 15%	CRYS—Saline	ECO—TT/TE	55
Jacquet-Lagrèze et al. [136]	2019	500	–	20	25	CI ≥ 15%	CRYS—Ringer A/L	CAL—PiCCO	38
Beurton et al. [138]	2019	500	–	10	50	CI ≥ 15%	CRYS—Saline	CAL—PiCCO2	60
Roger et al. [140]	2019	500	–	10	50	SV ≥ 15%	CRYS—Ringer A/L	ECO—TT/TE	53
Mukhtar et al. [142]	2019	500	–	16	31	SV ≥ 15%	COLL—Albumine	ECO—TT/TE	68
Trifi et al. [143]	2019	500	–	15	33	SV ≥ 15%	CRYS—Saline	ECO—TT/TE	70
Giraud et al. [145]	2018	500	–	10	50	CO ≥ 15%	CRYS—Saline	CAL—PiCCO	45

COLL, colloids; CRYS, crystalloids; HES 6%, hydroxyethyl starch 6%; Ringer A\L, ringer acetate\lactate; CAL, thermodilution/chemodilution calibrated device; UNCAL, pulse wave analysis uncalibrated device; ABF, aortic blood flow; CO, cardiac output; CI, cardiac index; SV, stroke volume; SVI, stroke volume index; VTI, velocity-time integral; CardioQ, Deltex Medical Ltd, Chichester, UK; ECO-TT\TE, transthoracic\transoesophageal echocardiography; FLOWTRAC/VIGILEO, Edwards Lifescience Corporation, Irvine, Ca, USA; LIDCO, LIDCO group plc, London, UK; MOSTCARE, Pressure Recording Analytical Method, PRAM, Vytech Health®, Padova, Italy; NICOM, Non-Invasive Continuous Cardiac Output, Imedex, France; PAC, pulmonary artery catheter; PiCCO/ProAQT/PICCO2, PULSION Medical Systems; R, responders; Vol, volume

In 19 studies (15.3%), the volume of the fluid challenge was reported in mL/kg, with a median (IQR) of 7 (6–8) mL/kg (Table 2). A fixed volume of 500 mL was administered in 95 (76.6)% of the included studies. The median (IQR) of the dispensed volume of fluid was 500 (500–500) mL, infused in a median (IQR) of 18 (11–30) min. Then, the median (IQR) infusion rate was 25 (17–33) mL/min.

CO/CI was used as target variables in 78 (62.9%) studies, while SV/SVI was used in 40 (32.2%) studies. The other six studies (4.8%) adopted SV surrogates (ABF in 4 studies and VTI in two studies). In 88 (70.9%) studies, the threshold adopted to define the fluid responsiveness was an increase of the considered variable ≥ 15% from baseline (Table 2).

Three studies (2.4%) [25–27] did not report the type of fluid used for the fluid challenge. Among the others, crystalloids were used in 68 (56.6)% studies, colloids in 52 (43.3) %, and blood in one (0.8)% (Table 2).

The majority of the studies [49 (39.5%)] used transpulmonary thermodilution/dye dilution calibrated haemodynamic monitoring; 22 (17.7%) studies adopted the pulmonary artery catheter monitoring. Echocardiography (either transthoracic or transoesophageal) was used in 31 (25.0)% of studies, and 5 (4.0%) used oesophageal doppler monitoring. Uncalibrated pulse wave analysis monitoring was used in the other 14 (11.2)% studies (Table 2). Finally, bioreactance was adopted in three studies (2.4%). Haemodynamic pre–post-fluid challenge variables in responders and non-responders populations are reported in Table 3.

**Table 3:** Haemodynamic parameters before and after fluid challenge administration in responders and non-responders

Haemodynamic variable	% DR	Pre FC	Post FC	% change pre versus post FC	*p* value–pre FC R versus NR	*p* value pre FC versus post FC
CI (L/min/m^2^)	44.3					
R		2.8 (2.5–3.2)	3.6 (3.0–4.1)	29 (23–33)	0.0003	< 0.0001
NR		3.3 (2.7–3.6)	3.4 (2.9–3.7)	5 (0–6)		0.09
SVI (ml/m^2^)	22.6					
R		29 (26–33)	39 (36–42)	29 (25–38)	0.0001	< 0.0001
NR		36 (31–41)	37 (31–42)	3 (− 1; 7)		0.05
MAP (mmHg)	73.4					
R		70 (68–74)	82 (77–85)	14 (10–18)	0.005	< 0.0001
NR		74 (70–80)	78 (75–85)	6 (4–8)		< 0.0001
SAP (mmHg)	33.9					
R		104 (99–108)	123 (113–129)	17 (12–22)	0.002	< 0.0001
NR		109 (105–118)	116 (109–123)	5 (4–8)		< 0.0001
PAOP (mmHg)	14.5					
R		11 (10–12)	15 (12–16)	28 (18–45)	0.05	< 0.0001
NR		13 (10–14)	16 (13–18)	28 (15–37)		0.005
CVP (mmHg)	42.7					
R		9 (7–11)	11 (10–13)	30 (19–41)	0.03	< 0.0001
NR		10 (8–12)	13 (10–15)	26 (15–38)		< 0.0001
HR (beats/min)	75.8					
R		98 (88–105)	94 (86–101)	− 3 (− 4; − 1)	0.03	< 0.0001
NR		94 (86–101)	91 (84–98)	− 2 (− 3; − 1)		< 0.0001
PPV (%)	30.6					
R		15 (12–18)	9 (5–11)	− 42 (− 53; − 29)	< 0.0001	< 0.0001
NR		8 (6–10)	7 (5–9)	− 15 (− 28; 0)		0.0002
SVV (%)	14.5					
R		14 (12–17)	10 (6–12)	− 36 (− 45; − 30)	< 0.0001	0.0002
NR		11 (8–13)	9 (6–11)	− 22 (− 33; − 9)		0.002

Data are presented as median (25th–75th interquartile) in responders (R) and non-responders (NR), n = 21; FC, fluid challenge; CI, cardiac index; SVI, stroke volume index; MAP, mean arterial pressure; SAP, systolic pressure variation; HR, heart rate; PPV, pulse pressure variation; SVV, stroke volume variation, CVP, central venous pressure; PAOP, pulmonary artery occluded pressure; %DR, percentage of data reported in the studies

### Trigger of fluid challenge administration.

Hypotension (i.e., systolic or mean arterial pressure below a fixed value or reduced by a fixed percentage from baseline) was used in 68 (62.4)% of studies. Oliguria (i.e. a drop in urine output below 0.5 mL/h for 2 or 3 consecutive hours) was used in 54 (49.5)% studies, skin mottling or peripheral hypoperfusion in 47 (43.1)% studies, tachycardia (i.e. an increase in heart rate above 100–110 beats/min) in 43 (39.4)%, the need for initiating the infusion or reducing the dose of vasoactive drugs in 41 (37.6)% studies, an increase in blood lactate in 34 (31.2)% studies, a diagnosis of sepsis/septic shock in 12 (11.0)% studies, and renal or hepatic dysfunction in seven (6.4)% studies. Fifteen studies (12.1%) did not report any trigger to start fluid challenge administration.

### Comparison of publication periods 2011–2021 versus 2000–2010

**Fig. 2:**
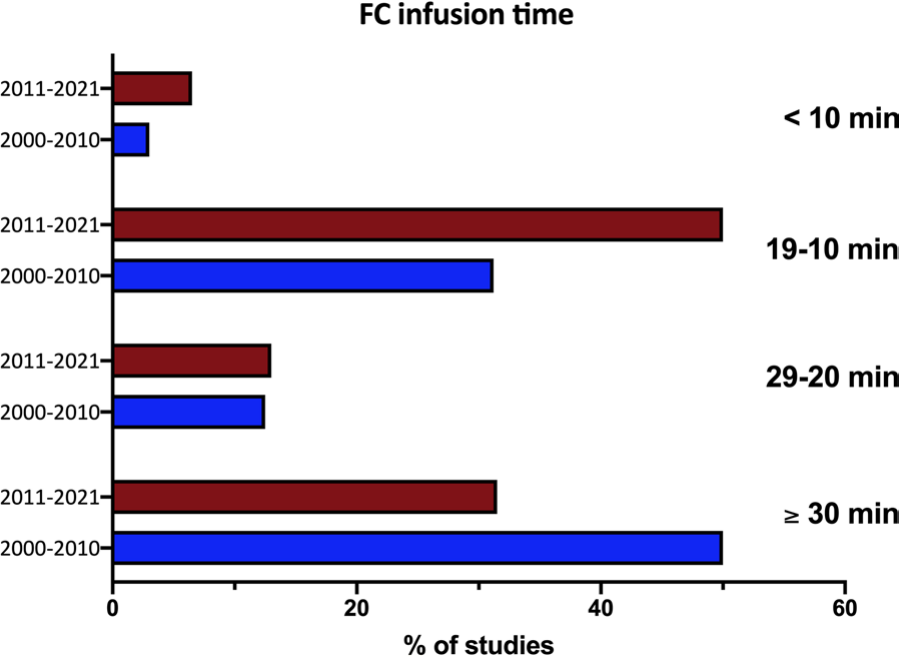
Percentage of studies in the two decades adopting different infusion timings. Fluid challenge, fluid challenge

The comparison between the 2000–2010 and 2011–2021 decades is reported in Table 4. The percentage of fluid responders (52% for both the decades) and the volume infused (500 mL) were comparable. On the contrary, the infusion time was lower in the last decade (a median of 15 (10–30) min vs 30 (15–30) min, *p* = 0.03). Crystalloids were used in 61.9% of studies published between 2011–2021 and 34.3% in the 2000–2010 decade (*p* = 0.007) (Figs. 2 and S1 in the Additional file 1﻿).

**Table 4:** Comparison between 2011–2021 and 2000–2010 decades regarding the modality of fluid challenge administration

General characteristics	2011–2021	2000–2010	*p* value
Fluid responders (%)	52 (45–60)	52 (43–62)	0.32
Crystalloids versus colloids (n. of studies)	57 versus 32	11 versus 20	0.007
Volume (ml)	500 (500–500)	500 (500–500)	0.32
Time of infusion (min)	15 (10–30)	30 (15–30)	0.03
Threshold 10% versus 15% (n. of studies)	30 versus 62	6 versus 26	0.17
CO/CI versus SV/SVI (n. of studies)	61 versus 30	18 versus 12	0.51

CO, cardiac output; CI, cardiac index; SV, stroke volume; SVI, stroke volume index

CO/CI was used in 67% of the studies published in 2011–2021 and in 60% of those published in 2000–2010 (*p* = 0.51). The threshold adopted was an increase in CO or surrogates ≥ 15% in 67.4% of the studies of the 2011–2021 decade and in 81.2% of the studies published in 2000–2010 (*p* = 0.17) (Additional file 1: Figure S1)﻿.

## Discussion

The results of this review, including research studies investigating the fluid challenge effect in critically ill adult patients receiving haemodynamic monitoring, may be summarised as follows: 1) fluid challenge is usually performed infusing a bolus of 500 mL of fluid, most often a crystalloid, in less than 20 min; 2) the response to fluid challenge is usually defined as a CI or CO increase ≥ 15% as compared to baseline; 3) positive response to fluid challenge is reported in about 50% of ICU patients; 4) the most common trigger for fluid challenge administration is usually the occurrence of hypotension, followed by oliguria and clinical signs of hypoperfusion; 5) the comparison between the 2000–2010 and 2011–2021 decades of publication showed no difference in the percentage of fluid responders (52% on average for both the decades), the volume infused (500 ml), and the criteria defining fluid responsiveness. On the contrary, compared to the 2000–2010 decade, in the period 2011–2021, the fluid challenge infusion time was lower, and crystalloids were more frequently used.

### Fluid challenge characteristics

Among the included studies, the fluid challenge usually consisted of a median volume of 500 mL administered over a 20-min period and defined as a positive response by an increase ≥ 15% of CO or surrogate. These characteristics and responsiveness definition are to be considered good practice, for the response of CO to a fluid bolus is poorly followed by the simultaneous changes in arterial pressure [28, 29] or heart rate [30]. However, this is not the case in clinical practice, where the fluid challenge effect is often assessed by a rise in arterial blood pressure [16].

Interestingly, 500 mL was also the median volume fluid challenge used in the FENICE study (an observational study including 311 centres across 46 countries) [16], whereas a fluid challenge of 250 mL is usually adopted in high-risk surgical patients undergoing goal-directed therapy optimisation [31]. The use of large volumes for fluid challenge optimisation should be balanced to the detrimental risk of fluid overload [9], primarily if safety limits (i.e.*,* increase in CVP) dynamically indicate fluid non-responsiveness are rarely used [19]. Since fluid challenge volume should be at least 4 mL/kg [32], smaller fluid challenge volumes may be considered for repetitive tests.

Moreover, the FENICE study reported a median of 24 min of infusion time and a rate of 17 mL/min [16]. Hence, the volume and rate of administration seem comparable between clinical and research settings. On the contrary, the infusion time was lower in the last decade (a median of 15 min vs 30 min, *p* = 0.03), indicating a trend towards the increase in the infusion rate in more recent studies. This global inception cohort study evaluated the clinical use of the fluid challenge in daily practice, whereas our review considered only research papers adopting the fluid challenge as a part of a protocol, limiting the comparison with the results of the FENICE. Moreover, in contrast with a previous metanalysis, including ICU studies up to 2014 [19], crystalloids are used in most studies. Crystalloids have been used in two-thirds of the studies from 2011 to 2021, compared to one-third from 2000 to 2010. These data indicate an alignment between research studies, recent guidelines, and metanalyses [13, 14].

### Limitations

Limitations of our review have to be considered when extrapolating the results to clinical practice. First, the present study does not report any outcome endpoints. A recent large randomised-controlled trial showed no difference in mortality rate among ICU patients receiving different fluid bolus infusion rates [33]. However, the faster rate adopted in this study (5.5 mL/min) is below the median rate found in the studies included in the present review (25 mL/min) [33]. The administration of aliquots of fluids at a slow rate should not probably be indicated as a fluid challenge. Moreover, all the included studies are research papers whose aim was to evaluate the haemodynamic changes after the fluid challenge infusion or assess the reliability of indexes or functional haemodynamic tests in predicting the response to a fluid challenge. We did not include studies on the fluid challenge clinical use in ICU patients.

Another potential source of bias is related to the different haemodynamic monitorings used to assess fluid challenge responsiveness. When considering the median cut-off value identifying responders from non-responders, the accuracy of measurement of the changes in CO, or its surrogates, is undoubtedly relevant. Additionally, the reliability of different monitorings in tracking the dynamic trends of CO may not be consistent and may be below the boundaries of accuracy and precision of the Critchley–Critchley criteria [34]. Hence, the reproducibility of CO measurements obtained by the different monitoring systems may be limited. Moreover, cut-off values and measurement techniques potentially induce heterogeneity in response to the fluid challenge administration. As confirmed, responders ranged from 23 to 100% across the included studies (Table 2). The use of echocardiography is associated with high proportions of fluid responders compared to other haemodynamic monitoring devices. The operator-dependent bias may affect the evaluation of SV changes after fluid challenge.

We excluded studies in which the fluid challenge response has been assessed without haemodynamic monitoring and, hence assessing changes in systemic arterial pressures, potentially limiting the whole comparability of the technique in the two considered decades. Finally, the overall number of patients enrolled in the trials of the two considered decades was considerably different. This could bias the comparisons between the two groups if weighted for study size.

## Conclusions

This systematic review, including research studies on fluid challenge use in critically ill adult patients receiving haemodynamic monitoring, showed a positive response in 52% of the patients. This test was usually performed infusing a bolus of 500 mL fluid, more often a crystalloid, in less than 20 min, and fluid responsiveness was generally indicated as a CI or CO increase ≥ 15% compared to baseline. Fluid challenge administration is usually triggered by hypotension. In the 2011–2021, the infusion time was shorter, and crystalloids were more frequently used than in the 2000–2010 decade.

## Supplementary Information

**Additional file 1**. **Table S1.** PRISMA-DTA checklist. **Table S2.** Extracted data in each study assessed for eligibility. **Table S3.** Full-text articles excluded, not fitting eligibility criteria. **Table S4.** Studies on functional haemodynamic tests or dynamic indexes of fluid responsiveness. **Figure S1.** Characteristics of fluid challenge administration and monitoring along the considered years.

## Data Availability

The datasets used and/or analysed during the current study are available from the corresponding author on reasonable request.

## Abbreviations

CI: Cardiac index
SVI: Stroke volume index
CO: Cardiac output
SV: Stroke volume
ICU: Intensive care unit
CVP: Central venous pressure
VTI: Velocity–time integral in the left ventricular outflow tract
ABF: Aortic blood flow
