# Supplementary material for: Fluid challenge in critically ill patients receiving haemodynamic monitoring: a systematic review and comparison of two decades
Source: Crit Care. 2022 Jun 21;26:186. doi: 10.1186/s13054-022-04056-3 (PMC9210670; doi:10.1186/s13054-022-04056-3)
Supplement: Supplementary file 1 — Additional file 1. Table S1. PRISMA-DTA checklist. Table S2. Extracted data in each study assessed for eligibility. Table S3. Full-text articles excluded, not fitting eligibility criteria. Table S4. Studies on functional haemodynamic tests or dynamic indexes of fluid responsiveness. Figure S1. Characteristics of fluid challenge administration and monitoring along the considered years. [file 13054_2022_4056_MOESM1_ESM.docx]

**Fluid challenge in critically ill patients receiving haemodynamic monitoring: a systematic review and comparison of two decades.**

Running head: Fluid challenge in ICU

Antonio Messina, MD, PhD^1,2^; Lorenzo Calabrò, MD^1^; Luca Pugliese^1^, MD; Aulona Lulja^1^; Alexandra Sopuch^1^; Daniela Rosalba, MD^3^; Emanuela Morenghi^1,2^; Glenn Hernandez^4^; Xavier Monnet, MD^5,6,7^; Maurizio Cecconi, MD, FRCA, FICM^1,2^.

^1^Humanitas Clinical and Research Center – IRCCS, Rozzano (Milano), Italy; ^2^Department of Biomedical Sciences, Humanitas University, Pieve Emanuele, MI, Italy;^3^Università del Piemonte Orientale, Vercelli, Italy; ^4^Departamento de Medicina Intensiva, Facultad de Medicina, Pontificia Universidad Católica de Chile, Diagonal Paraguay 362, Santiago, Chile; ^5^Hôpitaux Universitaires Paris-Sud, Hôpital de Bicêtre, Medical Intensive Care Unit, Le Kremlin-Bicêtre, F-94270; ^6^Université Paris-Saclay, AP-HP, Service de médecine intensive-réanimation, Hôpital de Bicêtre; ^7^DMU CORREVE, Inserm UMR S_999, FHU SEPSIS, Groupe de recherche clinique CARMAS, Le Kremlin-Bicêtre, France.

**Corresponding author:**

Antonio Messina, MD, PhD. Department of Anesthesia and Intensive Care Medicine;

IRCCS Humanitas, Humanitas University; Via Alessandro Manzoni, 56; 20089 - Rozzano – Milano (Italy). Tel: +39(0)2 8224 1

email: [antonio.messina@humanitas.it](mailto:antonio.messina@humanitas.it)

**SUPPLEMENTAL MATERIALS**

**Table of Contents**

**Table S1** PRISMA-DTA checklist.

**Table S2** Extracted data in each study assessed for eligibility.

**Table S3** Full-text articles excluded, not fitting eligibility criteria.

**Table S4** Studies on functional haemodynamic tests or dynamic indexes of fluid responsiveness.

**Figure S1.** Characteristics of fluid challenge administration and monitoring along the considered years.

**Table S1. PRISMA-DTA checklist for systematic review and meta-analysis**

| **Section/topic** | **#** | **Checklist item** | **Reported on page #** |
| --- | --- | --- | --- |
| **TITLE** | | |  |
| Title | 1 | Identify the report as a systematic review, meta-analysis, or both. | 1 |
| **ABSTRACT** | | |  |
| Structured summary | 2 | Provide a structured summary including, as applicable: background; objectives; data sources; study eligibility criteria, participants, and interventions; study appraisal and synthesis methods; results; limitations; conclusions and implications of key findings; systematic review registration number. | 2 |
| **INTRODUCTION** | | |  |
| Rationale | 3 | Describe the rationale for the review in the context of what is already known. | 3-4 |
| Objectives | 4 | Provide an explicit statement of questions being addressed with reference to participants, interventions, comparisons, outcomes, and study design (PICOS). | 4-5 |
| **METHODS** | | |  |
| Protocol and registration | 5 | Indicate if a review protocol exists, if and where it can be accessed (e.g., Web address), and, if available, provide registration information including registration number. | 6 |
| Eligibility criteria | 6 | Specify study characteristics (e.g., PICOS, length of follow-up) and report characteristics (e.g., years considered, language, publication status) used as criteria for eligibility, giving rationale. | 6 |
| Information sources | 7 | Describe all information sources (e.g., databases with dates of coverage, contact with study authors to identify additional studies) in the search and date last searched. | 6 |
| Search | 8 | Present full electronic search strategy for at least one database, including any limits used, such that it could be repeated. | 6 |
| Study selection | 9 | State the process for selecting studies (i.e., screening, eligibility, included in systematic review, and, if applicable, included in the meta-analysis). | 6-7 |
| Data collection process | 10 | Describe method of data extraction from reports (e.g., piloted forms, independently, in duplicate) and any processes for obtaining and confirming data from investigators. | 6-7 |
| Data items | 11 | List and define all variables for which data were sought (e.g., PICOS, funding sources) and any assumptions and simplifications made. | 8 |
| Risk of bias in individual studies | 12 | Describe methods used for assessing risk of bias of individual studies (including specification of whether this was done at the study or outcome level), and how this information is to be used in any data synthesis. | 8 |
| Summary measures | 13 | State the principal summary measures (e.g., risk ratio, difference in means). | 7-8 |
| Synthesis of results | 14 | Describe the methods of handling data and combining results of studies, if done, including measures of consistency (e.g., I^2^) for each meta-analysis. | 7-8 |

Page 1 of 2

| **Section/topic** | **#** | **Checklist item** | **Reported on page #** |
| --- | --- | --- | --- |
| Risk of bias across studies | 15 | Specify any assessment of risk of bias that may affect the cumulative evidence (e.g., publication bias, selective reporting within studies). | NA |
| Additional analyses | 16 | Describe methods of additional analyses (e.g., sensitivity or subgroup analyses, meta-regression), if done, indicating which were pre-specified. | 7-8 |
| **RESULTS** | | |  |
| Study selection | 17 | Give numbers of studies screened, assessed for eligibility, and included in the review, with reasons for exclusions at each stage, ideally with a flow diagram. | 9 |
| Study characteristics | 18 | For each study, present characteristics for which data were extracted (e.g., study size, PICOS, follow-up period) and provide the citations. | 9 |
| Risk of bias within studies | 19 | Present data on risk of bias of each study and, if available, any outcome level assessment (see item 12). | NA |
| Results of individual studies | 20 | For all outcomes considered (benefits or harms), present, for each study: (a) simple summary data for each intervention group (b) effect estimates and confidence intervals, ideally with a forest plot. | 10-11 |
| Synthesis of results | 21 | Present results of each meta-analysis done, including confidence intervals and measures of consistency. | 10-11 |
| Risk of bias across studies | 22 | Present results of any assessment of risk of bias across studies (see Item 15). | NA |
| Additional analysis | 23 | Give results of additional analyses, if done (e.g., sensitivity or subgroup analyses, meta-regression [see Item 16]). | 10-11 |
| **DISCUSSION** | | |  |
| Summary of evidence | 24 | Summarize the main findings including the strength of evidence for each main outcome; consider their relevance to key groups (e.g., healthcare providers, users, and policy makers). | 12 |
| Limitations | 25 | Discuss limitations at study and outcome level (e.g., risk of bias), and at review-level (e.g., incomplete retrieval of identified research, reporting bias). | 13 |
| Conclusions | 26 | Provide a general interpretation of the results in the context of other evidence, and implications for future research. | 14 |
| **FUNDING** | | |  |
| Funding | 27 | Describe sources of funding for the systematic review and other support (e.g., supply of data); role of funders for the systematic review. | 15 |

**Table S2. Extracted data in each study assessed for eligibility.**

| Study Reference and Study design and type | Names and surnames of authors, year of publication; Country/countries in which the study was carried out; Setting where the study has been performed |
| --- | --- |
| Patients’ characteristics | Age; male/female ratio; Ventilatory setting; predictive scores; vasopressors and inotropes use at baseline; haemodynamic response to the fluid challenge |
| Fluid challenge characteristics | Volume, type of fluid, haemodynamic monitoring, rate of fluid responders; rate of fluid infusion. |
| Reason for fluid challenge infusion | Criteria of haemodynamic instability. |

**Table S3. Full text articles excluded, not fitting eligibility criteria.**

| The effect of fluid bolus administration on cerebral tissue oxygenation in post-cardiac arrest patients | Bogaerts E., Ferdinande B., Palmers P.J., Malbrain M.L.N.G., Van Regenmortel N., Wilmer A., Lemmens R., Janssens S., Nijst P., De Deyne C., Verhaert D., Mullens W., Dens J., Dupont M., Ameloot K. | 2021 | Fluid responsiveness cut-off not specified |
| --- | --- | --- | --- |
| Carotid Ultrasound in Assessing Fluid Responsiveness in Patients with Hypotension and Suspected Sepsis | Liteplo A.S., Schleifer J., Marill K.A., Huang C.K., Gouker S.K., Ratanski D., Diamond E., Filbin M.R., Shokoohi H. | 2021 | no monitoring device of CO |
| The effect of fluid bolus administration on cerebral tissue oxygenation in post-cardiac arrest patients | Bogaerts E., Ferdinande B., Palmers P.J., Malbrain M.L.N.G., Van Regenmortel N., Wilmer A., Lemmens R., Janssens S., Nijst P., | 2021 | Fluid responsiveness cut-off not specified |
| Accuracy of a multiparametric score based on pulse wave analysis for prediction of fluid responsiveness: ancillary analysis of an observational study | Neuschwander A., Barthélémy R., Ditchi D., Dramé F., Redouté M., Stern J., Cholley B., Mebazaa A., Chousterman B.G., Pirracchio R. | 2020 | Infusion rate not specified |
| The relation between common carotid artery diameter and central venous pressure for assessment of intravascular fluid status after major surgeries; an observational study | Rashwan S.A.K., Bassiouny A.A.E., Badawy A.A., Mohammed A.R. | 2020 | fluid challenge characteristics not stated |
| Haemodynamically stable oliguric patients usually do not respond to fluid challenge | Felice V.B., Lisboa T.C., Souza L.V., Sell L.C., Friedman G. | 2020 | Fluid responsiveness cut-off not specified |
| Dynamic arterial elastance for predicting mean arterial pressure responsiveness after fluid challenges in acute respiratory distress syndrome patients | Luetrakool P., Morakul S., Tangsujaritvijit V., Pisitsak C. | 2020 | no monitoring device of CO |
| Changes in Radial Artery Pulse Pressure During a Fluid Challenge Cannot Assess Fluid Responsiveness in Patients With Septic Shock | De la Puente-Diaz de Leon V., de Jesus Jaramillo-Rocha V., Teboul J.-L., Garcia-Miranda S., Martinez-Guerra B.A., Dominguez-Cherit G. | 2020 | no monitoring device of CO |
| Dynamic arterial elastance for predicting mean arterial pressure responsiveness after fluid challenges in acute respiratory distress syndrome patients | Luetrakool P., Morakul S., Tangsujaritvijit V., Pisitsak C. | 2020 | no monitoring device of CO |
| Changes in Radial Artery Pulse Pressure During a Fluid Challenge Cannot Assess Fluid Responsiveness in Patients With Septic Shock | De la Puente-Diaz de Leon V., de Jesus Jaramillo-Rocha V., Teboul J.-L., Garcia-Miranda S., Martinez-Guerra B.A., Dominguez-Cherit G. | 2020 | no monitoring device of CO |
| Influence of changes in ventricular systolic function and loading conditions on pulse contour analysis-derived femoral dP/dt (max) | Vaquer S., Chemla D., Teboul J.-L., Ahmad U., Cipriani F., Oliva J.C., Ochagavia A., Artigas A., Baigorri F., Monnet X. | 2019 | Fluid responsiveness cut-off not specified |
| Influence of systemic haemodynamics on microcirculation during sepsis | Collet M., Huot B., Barthélémy R., Damoisel C., Payen D., Mebazaa A., Chousterman B.G. | 2019 | Fluid responsiveness cut-off not specified |
| Volume expansion and variation in haemodynamic parameters | Martos-Benítez F.D., Guzmán-Breff B.I. | 2019 | Fluid responsiveness cut-off not specified |
| Monitoring haemodynamic response to fluid-challenge in ICU: Comparison of pressure recording analytical method and oesophageal Doppler: A prospective observational study | Barthélémy R., Neuschwander A., Dramé F., Redouté M., Ditchi D., Stern J., Mebazaa A., Pirracchio R., Chousterman B.G. | 2019 | Fluid responsiveness cut-off not specified |
| Carotid and femoral Doppler do not allow the assessment of passive leg raising effects | Girotto V., Teboul J.-L., Beurton A., Galarza L., Guedj T., Richard C., Monnet X. | 2018 | Fluid responsiveness cut-off not specified |
| Predictors, Prevalence, and Outcomes of Early Crystalloid Responsiveness Among Initially Hypotensive Patients With Sepsis and Septic Shock | Leisman D.E., Doerfler M.E., Schneider S.M., Masick K.D., D’Amore J.A., D’Angelo J.K. | 2018 | there is no valid monitoring device |
| Echocardiography and passive leg raising in the postoperative period: A prospective observational study | El Hadouti Y., Valencia L., Becerra A., Rodríguez-Pérez A., Vincent J.L. | 2017 | Infusion rate not specified |
| Noninvasive oscillometric cardiac output determination in the intensive care unit - comparison with invasive transpulmonary thermodilution | Reshetnik A., Compton F., Schölzel A., Tölle M., Zidek W., Giet M.V. | 2017 | Infusion rate not specified |
| Different effects of fluid loading with saline, gelatine, hydroxyethyl starch or albumin solutions on acid-base status in the critically ill | Spoelstra-de Ma A.M.E., Smorenberg A., Groeneveld A.B.J. | 2017 | Fluid responsiveness cut-off not specified |
| Adequate fluid resuscitation in septic shock with high catecholamine doses | Lewejohann J.C., Braasch H., Hansen M., Zimmermann C., Muhl E., Keck T. | 2016 | fluid challenge characteristics not stated |
| Preload dependence indices to titrate volume expansion during septic shock: A randomized controlled trial | Richard J.-C., Bayle F., Bourdin G., Leray V., Debord S., Delannoy B., Stoian A.C., Wallet F., Yonis H., Guerin C. | 2015 | Fluid responsiveness cut-off not specified |
| Effects of fluid administration on renal perfusion in critically ill patients |  | 2015 | no monitoring device of CO |
| Fluid responsiveness and brain tissue oxygen augmentation after subarachnoid haemorrhage | Kurtz P., Helbok R., Ko S.-B., Claassen J., Schmidt J.M., Fernandez L., Stuart R.M., Connolly E.S., Badjatia N., Mayer S.A., Lee K. | 2014 | Fluid responsiveness cut-off not specified |
| Jugular vein distensibility predicts fluid responsiveness in septic patients | Guarracino F., Ferro B., Forfori F., Bertini P., Magliacano L., Pinsky M.R. | 2014 | Fluid responsiveness cut-off not specified |
| Left ventricular torsion abnormalities in septic shock and corrective effect of volume loading: A pilot study | Bloechlinger S., Berger D., Bryner J., Wiegand J., Dünser M.W., Takala J. | 2013 | Fluid responsiveness cut-off not specified |
| The effects of propofol and dexmedetomidine infusion on fluid responsiveness in critically ill patients | Yu T., Huang Y., Guo F., Yang Y., Teboul J.-L., Qiu H. | 2013 | Fluid responsiveness cut-off not specified |
| Echocardiography and pulse contour analysis to assess cardiac output in trauma patients | Franchi F., Falciani E., Donadello K., Zacà V., Silvestri R., Taccone F.S., Cubattoli L., Mongelli P., Giomarelli P., Scolletta S. | 2013 | Fluid responsiveness cut-off not specified |
| Evaluation of cardiac output in intensive care using a non-invasive arterial pulse contour technique (Nexfin®) compared with echocardiography | Taton O., Fagnoul D., De Backer D., Vincent J.-L. | 2013 | Haemodynamic data not reported |
| Qualitative assessment of the inferior vena cava: Useful tool for the evaluation of fluid status in critically ill patients | Ferrada P., Anand R.J., Whelan J., Aboutanos M.A., Duane T., Malhotra A., Ivatury R. | 2012 | Fluid responsiveness cut-off not specified |
| Both passive leg raising and intravascular volume expansion improve sublingual microcirculatory perfusion in severe sepsis and septic shock patients | Pottecher J., Deruddre S., Teboul J.-L., Georger J.-F., Laplace C., Benhamou D., Vicaut E., Duranteau J. | 2010 | Fluid responsiveness cut-off not specified |
| Tracking hypotension and dynamic changes in arterial blood pressure with brachial cuff measurements | Lakhal K., Ehrmann S., Runge I., Legras A., Dequin P.-F., Mercier E., Wolff M., Régnier B., Boulain T. | 2009 | Infusion rate not specified |
| Haemodynamic effects of 6% and 10% hydroxyethyl starch solutions versus 4% albumin solution in septic patients | Friedman G., Jankowski S., Shahla M., Gomez J., Vincent J.-L. | 2008 | Fluid responsiveness cut-off not specified |
| Acute kidney injury criteria predict outcomes of critically ill patients | Barrantes F., Tian J., Vazquez R., Amoateng-Adjepong Y., Manthous C.A. | 2008 | Fluid responsiveness cut-off not specified |
| Relationship between the tricuspid annular plane systolic excursion and right and left ventricular function in critically ill patients | Lamia B., Teboul J.-L., Monnet X., Richard C., Chemla D. | 2007 | Infusion rate not specified |
| Changes in aortic blood flow induced by passive leg raising predict fluid responsiveness in critically ill patients | Lafanechère A., Pène F., Goulenok C., Delahaye A., Mallet V., Choukroun G., Chiche J.-D., Mira J.-P., Cariou A. | 2006 | Infusion rate not specified |
| Pre-ejection period variations predict the fluid responsiveness of septic ventilated patients | Feissel M., Badie J., Merlani P.G., Faller J.-P., Bendjelid K. | 2005 | Infusion rate not specified |

CO, cardiac output.

**Figure S1.**

Characteristics of fluid challenge administration and monitoring along the considered years.


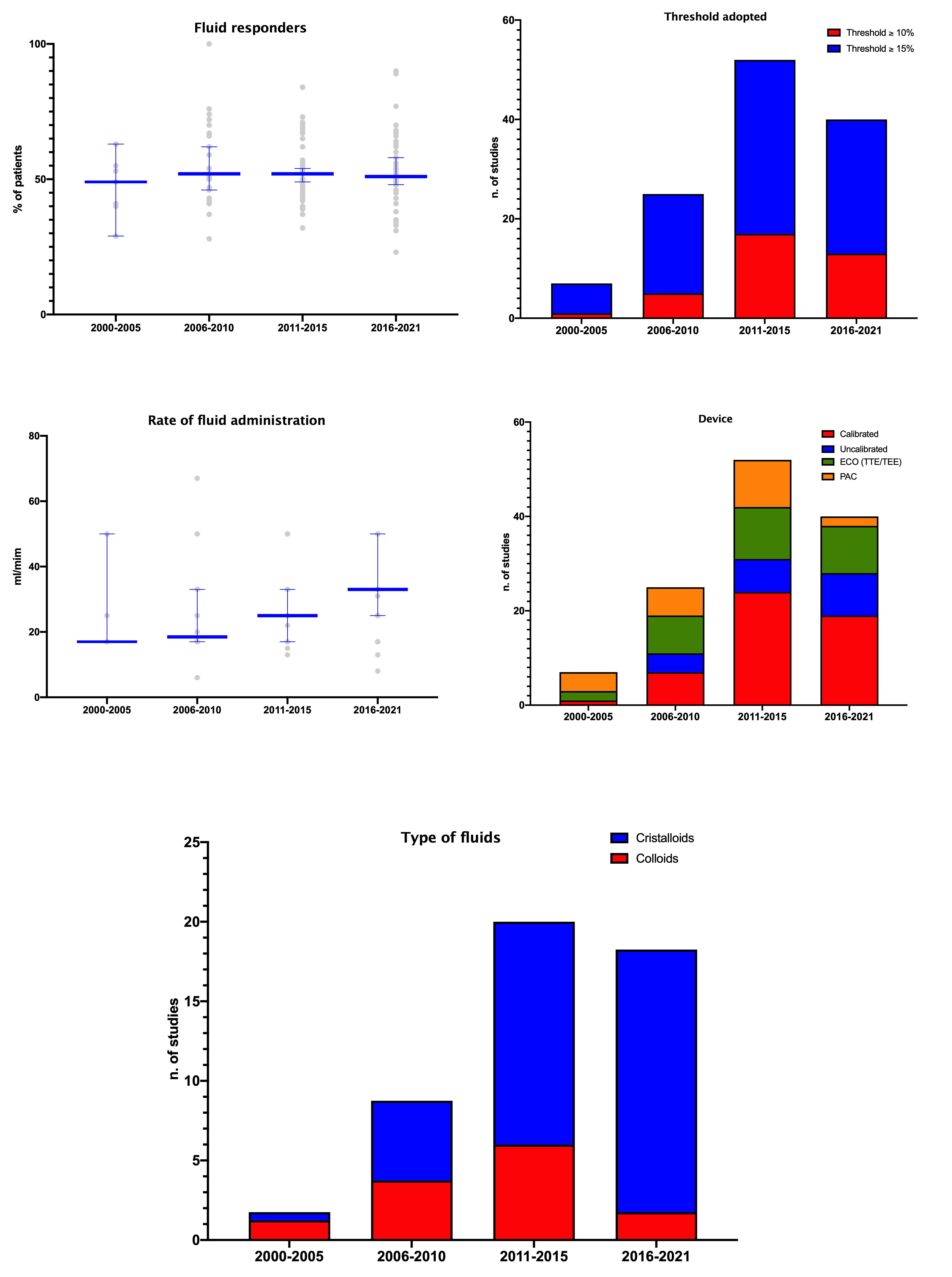


Figure S1: ECO, ecocardiography; TTE, transthoracic; TEE, transesophageal; PAC, pulmonary artery catheter.

ECO
